# Efficient identification of patients eligible for clinical studies using case-based reasoning on Scottish Health Research register (SHARE)

**DOI:** 10.1186/s12911-020-1091-6

**Published:** 2020-04-19

**Authors:** Wen Shi, Tom Kelsey, Frank Sullivan

**Affiliations:** 1 School of Medicine, University of St. Andrews, North Haugh, St. Andrews, Scotland KY16 9TF UK; 2 School of Computer Science, University of St. Andrews, North Haugh, St. Andrews, Scotland KY16 9SX UK

**Keywords:** Clinical studies, Electronic health record, Machine learning, Artificial intelligence

## Abstract

**Background:**

Trials often struggle to achieve their target sample size with only half doing so. Some researchers have turned to Electronic Health Records (EHRs), seeking a more efficient way of recruitment. The Scottish Health Research Register (SHARE) obtained patients’ consent for their EHRs to be used as a searching base from which researchers can find potential participants. However, due to the fact that EHR data is not complete, sufficient or accurate, a database search strategy may not generate the best case-finding result. The current study aims to evaluate the performance of a case-based reasoning method in identifying participants for population-based clinical studies recruiting through SHARE, and assess the difference between its resultant cohort and the original one deriving from searching EHRs.

**Methods:**

A case-based reasoning framework was applied to 119 participants in nine projects using two-fold cross-validation, with records from a further 86,292 individuals used for testing. A prediction score for study participation was derived from the diagnosis, procedure, pharmaceutical prescription, and laboratory test results attributes of each participant. Evaluation was conducted by calculating Area Under the ROC Curve and information retrieval metrics for the ranking list of the test set by prediction score. We compared the most likely participants as identified by searching a database to those ranked highest by our model.

**Results:**

The average ROCAUC for nine projects was 81% indicating strong predictive ability for these data. However, the derived ranking lists showed lower predictive performance, with only 21% of the persons ranked within top 50 positions being the same as identified by searching databases.

**Conclusions:**

Case-based reasoning is may be more effective than a database search strategy for participant identification for clinical studies using population EHRs. The lower performance of ranking lists derived from case-based reasoning means that patients identified as highly suitable for study participation may still not be recruited. This suggests that further study is needed into improvements in the collection and curation of population EHRs, such as use of free text data to aid reliable identification of people more likely to be recruited to clinical trials.

## Background

Paradoxically, Randomised Controlled Trials (RCTs) often fail because they fail to recruit sufficient study subjects, even when many potential study subjects would agree to participate [1]. They are often not asked to participate because of inefficiencies in recruitment processes. The recent Cochrane review conducted by Treweek et al. of 72 different recruitment strategies identified only three effective interventions with high certainty and another four with moderate certainty [2]. Electronic Health Records have recently become increasingly important sources for identifying trial participants [3–6].

A potential application of EHR data analysis is to identify prospective participants for forthcoming RCTs, thereby improving the recruitment and selection process. A 2015 study showed that careful analysis of EHR data could reliably identify individuals eligible to participate in clinical trials [7]. For each of 13 diverse clinical trials - each conducted at Columbia University - diagnostic, pharmacy, laboratory results and case notes from participants were used to derive a profile of an idealised target patient. Candidate participants were then matched in terms of conformity to this profile, based on their EHR data. The reported Area Under the ROC curve of 95% demonstrates the utility of both EHRs and the analytical approach taken. The problem remains of translating this approach to a population-based and multi-centre clinical study setting.

**Fig. 1:**
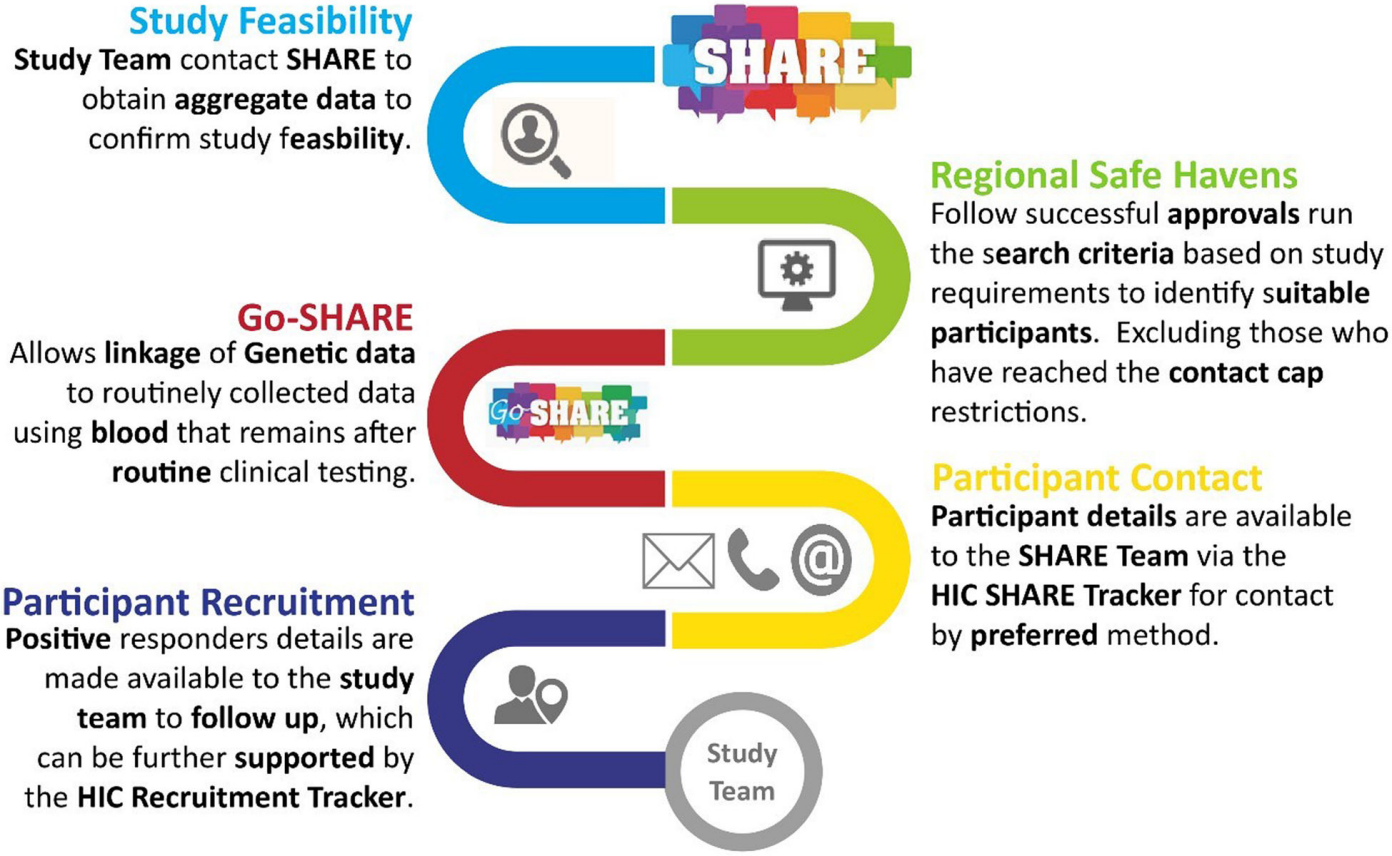
Work flow for recruiting participants to clinical studies through SHARE [11].

The Scottish Health Research Register (SHARE) [8] allows searching of linked EHR data for people using the Scottish National Health Service (NHS) who have opted in to allow access to their data for this purpose [9]. There are similar developments in other countries [10]. SHARE registrants are therefore population based, and can, in principle, be assessed as potential participants in clinical studies and other ethically approved, methodologically rigorous research. Scotland has 14 regional health boards delivering services to its 5.6 M citizens, and RCTs are conducted within and amongst any combination of boards. Figure 1 shows the workflow for the use of SHARE in recruitment of participants to forthcoming studies. Participant selection with SHARE is currently performed by searching the corresponding fields in the EHRs according to the elements contained in the study inclusion/exclusion criteria provided by the study researcher to the staff in the Health Informatics Centre: University of Dundee (HIC) [11]. This approach may fail to identify suitable participants due to incomplete and/or insufficient EHR data.

In this paper we investigate whether case-based reasoning, i.e. the use or adaptation of existing solutions may be applied to new problem instances [7], can generate a good predictive performance in participant identification for multi-centre and population-based studies in the Scottish EHR setting.

## Methods

The objective of this study is to: (1) evaluate the predictive performance of case-based reasoning (CBR) in studies conducted in a multi-centre and population-based manner using a range of different EHRs, (2) assess the consistency of participant prediction results between CBR and the database search strategy.

**Fig. 2:**
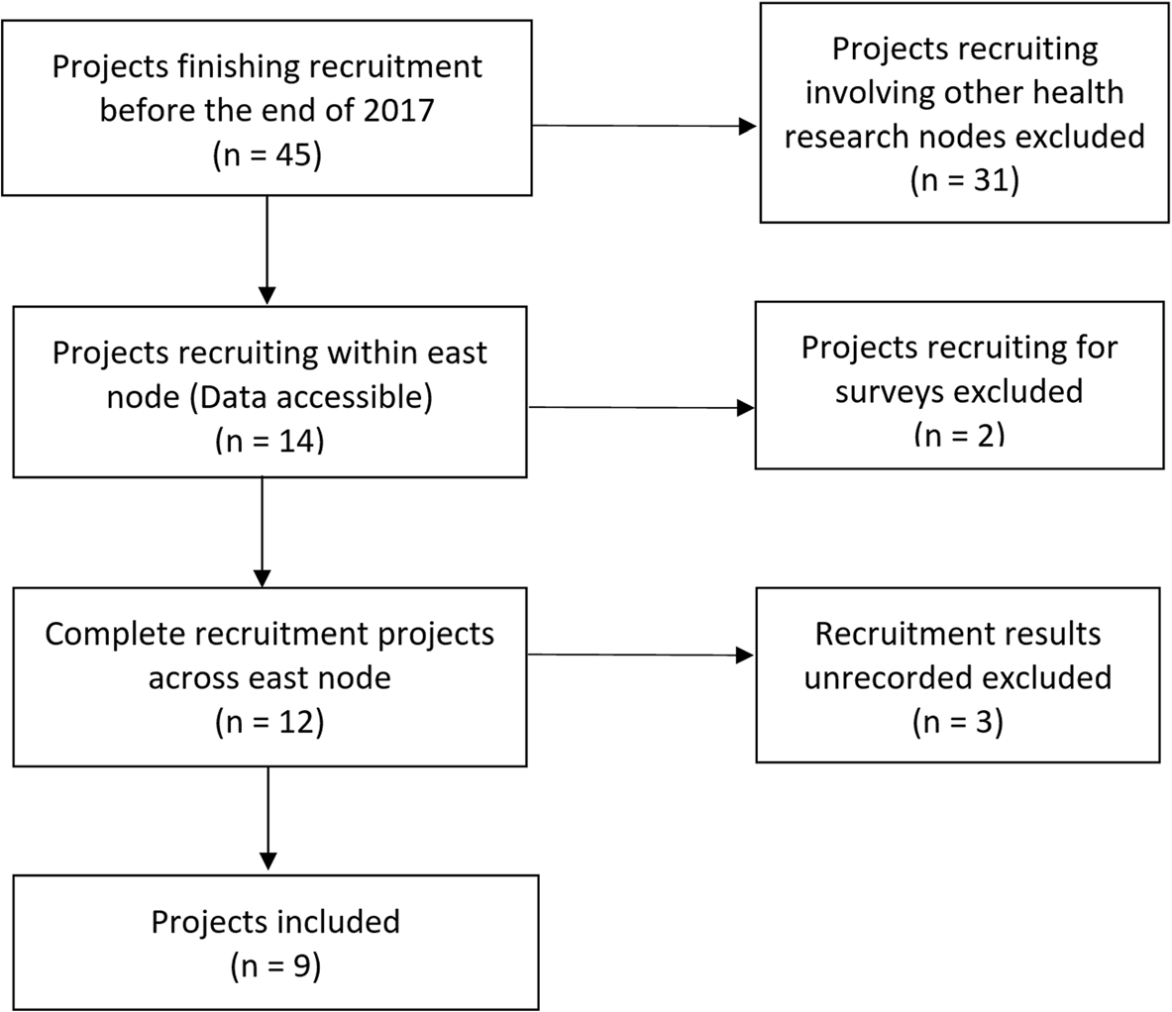
Flow diagram for the selection of projects for analysis

The current study included nine completed projects from SHARE for analysis. The process of inclusion is described in Fig. 2. These projects had completed recruitment before the end of 2017. They recruited participants across the health boards in Eastern Scotland. Finally, three projects without recruitment data recorded were also excluded.

The additional table (see additional file 1) summarizes the inclusion/exclusion criteria, number of participants required by the nine projects and the number of potential participants identified by searching database, the number of persons finally recruited in each project and how many of them were identified in the database. The projects involved from 2 to 28 participants, with no participant being recruited into more than one project. A total of 119 people were enrolled in these nine projects. We chose the individual final recruitment status recorded in SHARE as our gold standard to train and evaluate our algorithm since (i) we can compare our evaluation results with those from the original study because Miotto & Weng also used the final enrolment results as gold standard, and (ii) it is almost impossible to confirm the real eligibility of each person and use them as gold standard since this is a retrospective study.

The Community Health Index (CHI) is a population register, which is used in Scotland for healthcare purposes [12]. A CHI number uniquely identifies a person on the index. The data from selected projects were de-identified by removing personal data such as names, addresses and date of birth, and linked through CHI number for each participant. Recruitment data were extracted and compiled by HIC, composed of persons identified and recruited for each of the nine projects. Clinical data – diagnosis, procedure, prescription and laboratory test were requested for all 119 participants and all other registrants of SHARE in the NHS Scotland Fife and Tayside areas (*n* = 90,456). Following assessment of the time frames of the projects to be analysed and the availability and completeness of the clinical data needed, the requested clinical data range from 2010 through 2017. Diagnoses were recorded using the International Classification of Diseases revision 10 (ICD-10). Procedures were recorded using the Office of Population Censuses and Surveys, Classification of Surgical Operations and Procedures version 4 (OPCS-4). Prescriptions were recorded using unique identifiers mapped to British National Formulary (BNF) codes [13] to identify the drug prescribed. Prescription data also included the quantities of each prescription dispensed. Finally, laboratory test data consisted of test names, read codes allocated to the specific test, and test results. Data were accessed and analysed through the data safe haven provided by HIC [14, 15].

Diagnoses and procedures were presented in entity-attribute-value (EAV) model [16, 17] in wide form in which one record of a certain person contains several diagnoses and procedures, the numbers of which vary from person to person. For example, patient A’s first record has diagnoses of pneumonia, diabetes, hypertension. These data were first transformed into a long EAV form with one record comprising one diagnosis or procedure alone. Prescription data were mapped to formatted BNF codes and the records with unidentifiable maps were excluded [18]. To ensure that each person included for analysis had some meaningful clinical data recorded, individuals with no diagnosis or procedure or prescription or lab test were excluded, as were those subjects having test results but incomplete test description. As a result, 119 participants having at least one record in diagnosis, procedure, prescription and lab test were included for further analysis. Eight six thousand two hundred ninety-two other registrants were included for use in test datasets for model assessment and validation purposes, being the subset of the 90,456 SHARE subjects that met the same inclusion criteria as the 119 participants.

Each project was analysed separately by two-fold cross-validation. For both folds, half of the 119 participants were used to train a predictive model. The remaining half were combined with (i) 30,000 SHARE registrants chosen randomly from the 86,292 available and (ii) all subjects identified though inclusion/exclusion database queries for the project but not selected as one of our 119 cases (people enrolled in studies). This test dataset was used to assess prediction errors for subjects not used to derive the model. Following the methodology of Miotto and Weng, 2015 [7], a target profile was derived from central tendencies of the occurrences of the codes from diagnosis, procedure, prescription and laboratory test. The featured codes were determined according to their frequencies among that project’s participants, with each entity (being either a diagnosis, a procedure, a prescription or a laboratory test) requiring a minimum of 10 codes shared by at least 80% of the participants. If more than 10 codes were retrieved in one entity, all the codes exceeding 80% threshold were included. If the data were sparse, with no code reaching 80%, all the codes were retained. After the target profile had been produced, for every individual a similarity score was produced for each of the four entities respectively by being compared against the target using cosine similarity, which uses orientation of entities when plotted in multi-dimensional space to assess similarity rather than magnitude of difference apart in the same space [19]. Thus, each person was represented by four similarity scores ranging from 0 to 1. The training set was used to train a linear regression model to get four optimal weights for each entity, which were then applied to each individual in the test set to obtain a final score which was then scaled to fall between 0 and 1.

For each project, the area under the Reciever-Observer Characteristic curve (ROCAUC) was calculated for either testing fold respectively, and average scores were obtained for projects individually and for the nine projects as a whole. ROCAUC measures the overall performance of a binary classifier [20]. It estimates the probability of obtaining a higher score for a participant than for a non-participant. The higher the ROCAUC, the better the classifier. For this study ROCAUC are interpreted using a standard quality scheme as follows: 90–100 = excellent; 80–90 = good; 70–80 = fair; 60–70 = poor; 50–60 = fail [21]. The confidence interval (CI) for ROCAUC was generated through 2000 bootstrap replicates [22]. Prediction results were also combined to identify the cut-off maximizing the performance metric that sums up sensitivity and specificity.

A ranking list was generated for each test set, with individual final scores decreasing for assessment with regard to their ability to find the person recruited using metrics such as precision of predicting the 5 top items (P5) [23], precision of predicting the 10 top items (P10) [23], the mean average precision (MAP) [24] and mean reciprocal rank (MRR) [25]. Average precision combines the precisions obtained every time a relevant result is retrieved, thereby assessing the quality of the whole list. Reciprocal rank is the reciprocal of the rank of the first targeted person retrieved, reflecting the utility of the list to meet the need of the list users when identifying suitable participants. The best possible result for the list with participants ranked at the top (Upper) and the worst result - a random list (Lower) were also obtained as references. The metrics were averaged across all 18 (i.e. 2 fold for each of 9 projects) ranking lists respectively.

We additionally examined how many of the persons identified by database queries were among the top 50 of the ranking list in proportion.

## Results

The ROCAUC for each project and each fold are shown in Table 1. The mean ROCAUC for prediction score was 0.815 over nine projects. One project had unacceptably low ROCAUC for prediction score (0.337) being worse than a random guess, with another project giving poor discriminatory performance (ROCAUC 0.619) The remaining eight projects were either good or excellent in terms of ROCAUC.

**Table 1:** The Area Under the ROC Curve for recruitment prediction test for each project. For each project the ROCAUC and 95% confidence intervals are given for the first and second cross validation datasets, followed by the average

Project Acronym	Fold 1 (CI)	Fold 2 (CI)	Average (CI)
ALPHA	0.947 (0.897–0.984)	0.855 (0.777–0.928)	0.901 (0.837–0.956)
ALLAY	0.928 (0.903–0.955)	0.854 (0.768–0.941)	0.891 (0.836–0.948)
METFORMIN	0.977 (0.970–0.989)	0.980 (0.970–0.983)	0.979 (0.970–0.986)
REFORM	0.946 (0.896–0.980)	0.957 (0.920–0.987)	0.952 (0.908–0.984)
IMPOCT	0.397 (NA)	0.276 (NA)	0.337 (NA)
TARDIS	0.897 (0.844–0.946)	0.887 (0.852–0.919)	0.892 (0.848–0.933)
4P	0.952 (0.909–0.993)	0.744 (0.447–0.978)	0.848 (0.678–0.986)
HF	0.907 (0.851–0.957)	0.927 (0.870–0.974)	0.917 (0.861–0.966)
ImmunoStat	0.632 (0.503–0.763)	0.605 (0.443–0.756)	0.619 (0.473–0.760)

NA: insufficient data to calculate an accurate CI

**Fig. 3:**
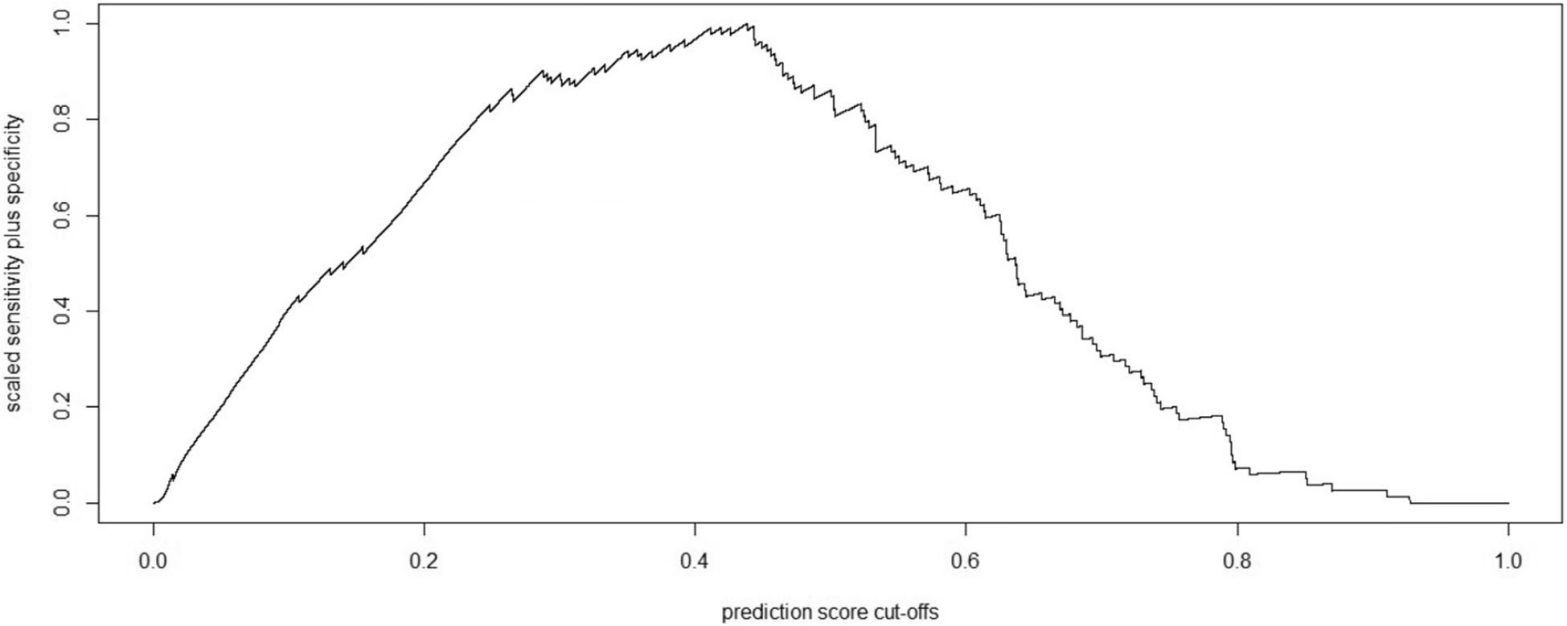
Scaled sensitivity plus specificity against prediction score cut-offs

A threshold of 0.44 gives the maximum performance metric for these data. A plot of performance against different prediction score cut-offs is shown in Fig. 3; the scaled sum of sensitivity and specificity was calculated for precision score thresholds from zero to 100%, with the optimal cut-off for these data occurring at 44%.

Ranking list results are shown in Table 2. Overall, the ranking list failed to present the relevant participants at neither the top 5 positions nor the top 10. Performance improved when trying to find participants from the top of the list of roughly 30,000 patients compared to a random list, but was still far from optimal.

**Table 2:** Performance of the resulted ranking lists. For each project we give the number of participants appearing in the top 5 and top 10 of a ranking list, the mean average precision (MAP) and the mean reciprocal rank (MRR)

List	P5	P10	MAP	MRR
ALPHA	0	0	0.003	0.006
ALLAY	0	0	0.001	0.001
METFORMIN	0	0	0.003	0.002
REFORM	0	0	0.010	0.035
IMPOCT	0	0	0.000	0.000
TARDIS	0	0	0.002	0.005
4P	0	0	0.002	0.003
HF	0	0	0.006	0.028
ImmunoStat	0	0	0.001	0.007
Average of all ranking lists	0	0	0.003	0.010
Averaged Lower	0	0	0.000	0.000
Averaged Upper	0.789	0.611	1	1

On average, 21% of the persons ranked within the top 50 positions on the ranking list were the same as identified by searching databases. The proportions of those both identified listed in the top 50 of the list for each project are presented in Table 3.

**Table 3:** Proportion of persons both identified within the top 50 positions of the ranking list for each project

Project acronym	Fold 1	Fold 2	Average
ALPHA	0.66	0.32	0.49
ALLAY	0.54	0.54	0.54
METFORMIN	0	0	0.00
REFORM	0.32	0.16	0.24
IMPOCT	0	0	0.00
TARDIS	0.22	0	0.11
4P	0.06	0.14	0.10
HF	0.30	0.42	0.36
ImmunoStat	0.04	0.08	0.06

## Discussion

Our ROCAUC results indicate that we can reliably and retrospectively predict the recruitment status for seven of the nine projects analysed, with IMPOCT and ImmunoStat being harder to predict in terms of participation. We speculate that, for IMPOCT study, the poor result was caused by only two participants being recruited, so that only one person was used to derive the target profile of participants. Considering the relatively loose inclusion/exclusion criteria for ImmunoStat, this less satisfactory performance may result from an inherent inability of capturing a representative target due to scattered participant characteristics. For the higher-quality projects, ROCAUC is either good (i.e. above 80%) or excellent (i.e. above 90%), providing strong support for our central claim that careful analysis of EHR data can reliably discriminate participation status for projects of sufficiently high quality.

Evaluation of the resultant ranking lists showed poor performance in retrieving the real participants at the top. None of the ranking lists identified participants at either the first five individuals retrieved or the first 10. The ranking performance was the worst for IMPOCT project, consistent with the AUC result. Performance improves slightly when identifying persons recruited and selecting the first candidate for participation, but is still unsatisfactory in terms of matching predicted to actual participants.

The assessment of the consistency of patients identified by case-based reasoning and database query shows there is significant discrepancy among the potential participants detected by the two methods. However, it is unknown whether the most likely participants identified from our ranking lists would have been recruited or not due to the retrospective nature of our study, so there is an inherent lack of discriminative ability involved. Also the list of eligible participants derived from searching the database is definitely not exhaustive. As shown in the project summary table (see additional file 1), a few of the participants were not among the persons identified in database at all. Therefore, the non-participants picked up by the ranking list who were not identified before might have been actual participants.

Our study has the following strengths: we have used recruitment data from real clinical studies involving multiple health boards and multiple data sources; the EHR data is population based and hence is not flawed by selection or geographic bias; we have used an analogue of an existing proven methodology augmented with cross validation and relative ranking analysis; we have compared the potential participants between two detecting strategies.

Our study also has several limitations. We acknowledge that the final recruitment results in SHARE are not a perfect gold standard because they are affected by some external factors rather than clinical information alone such as personal preferences. The algorithm might have correctly identified an eligible participant but this person had not been enrolled in the study due to personal reasons, although our evaluation results would only be underestimated in this case. Whilst acknowledging this inherent lack of knowledge as a limitation of the study, we believe that we have demonstrated results in agreement with Miotto & Weng’s key finding that by matching EHR data of unseen patients to a target derived by case-based reasoning, we can estimate relevance to a forthcoming trial, with higher relevance being associated with higher eligibility. Additionally, the clinical data used for this study do not consist of free-text clinical notes, possibly impacting on performance; a final limitation is that despite our careful checking of final recruitment status for each project, the recruitment data may contain some misclassifications.

## Conclusions

In this study we have demonstrated that case-based reasoning performed well in predicting recruitment for population-based clinical studies using Scottish EHR data. Our results were substantially worse than those reported by Miotto & Weng [7] with regard to ranking lists derived from the similarity scores from case-based reasoning analysis. Our results suggest that the provision of more accurate recruitment data and more abundant clinical data resources (including narrative clinical notes) would improve our ability to derive accurate ranking lists, with no negative impact on clinical care for the population (consistent with the primary purpose of EHRs). Planned prospective studies comparing the actual recruitment results for persons identified by differed methods should help address the question of which ones are most suitable for finding potential participants. Our analytical framework could usefully enhance the existing database query method in Scotland, and form the basis of participant prediction schemes in other healthcare settings.

## Supplementary information

**Additional file 1.** Summary of nine projects included for analysis.

## Data Availability

The study only has online access to the data stored in safe haven. Requests for data access should be directed to the Health Informatics Centre: University of Dundee.

## Abbreviations

BNF: British National Formulary
CBR: Case-based reasoning
CHI: Community Health Index
CI: Confidence interval
EAV: Entity-attribute-value
EHRs: Electronic Health Records
HIC: Health Informatics Centre: University of Dundee
ICD-10: International Classification of Diseases revision 10
MAP: Mean average precision
MRR: Mean reciprocal rank
NHS: National Health Service
OPCS-4: Office of Population Censuses and Surveys, Classification of Surgical Operations and Procedures version 4
P5: The 5 top items
P10: The 10 top items
RCTs: Randomised Controlled Trials
ROCAUC: Area Under the ROC Curve
SHARE: Scottish Health Research Register

## References

[1] Sully BGO, Julious SA, Nicholl J. A reinvestigation of recruitment to randomised, controlled, multicenter trials: a review of trials funded by two UK funding agencies. Trials. 2013;14(1). 10.1186/1745-6215-14-166. Accessed July 2019. [cited 2019 Jul]. 10.1186/1745-6215-14-166 PMC3691846 23758961

[2] Treweek S, Pitkethly M, Cook J, Fraser C, Mitchell E, Sullivan F, et al. Strategies to improve recruitment to randomised trials. Cochrane Database Syst Rev. 2018;2. 10.1002/14651858.MR000013.pub6. Accessed July 2019. [cited 2019 Jul]. 10.1002/14651858.MR000013.pub6 PMC7078793 29468635

[3] Wing K, Williamson E, Carpenter JR, Wise L, Schneeweiss S, Smeeth L, et al. Real-world effects of medications for chronic obstructive pulmonary disease: protocol for a UK population-based non-interventional cohort study with validation against randomised trial results. BMJ Open. 2018;8(3). Available from: http://bmjopen.bmj.com/content/8/3/e019475.long. Accessed July 2019. [cited 2019 Jul]. 10.1136/bmjopen-2017-019475. 10.1136/bmjopen-2017-019475 PMC5875594 29581202

[4] Staa TP, Goldacre B, Gulliford M, Cassell J, Pirmohamed M, Taweel A, et al. Pragmatic randomised trials using routine electronic health records: putting them to the test. BMJ. 2012;344. Available from: www.bmj.com/content/bmj/344/bmj.e55.full.pdf. Accessed July 2019. [cited Jul 2019]. 10.1136/bmj.e55. 10.1136/bmj.e55 PMC3934788 22315246

[5] James S, Rao SV, Granger CB. Registry-based randomized clinical trials—a new clinical trial paradigm. Nat Rev Cardiol. 2015;12(5). Available from: www.nature.com/articles/nrcardio.2015.33. Accessed July 2019. [cited Jul 2019]. 10.1038/nrcardio.2015.33. 10.1038/nrcardio.2015.33 25781411

[6] UK HARP-III Collaborative Group (2017). Randomized multicentre pilot study of sacubitril/valsartan versus irbesartan in patients with chronic kidney disease: United Kingdom heart and renal protection (HARP)- III-rationale, trial design and baseline data. Nephrol Dial Transplant.

[7] Miotto R, Weng C. Case-based reasoning using electronic health records efficiently identifies eligible patients for clinical trials. J Am Med Inform Assoc. 2015;22(e1). Available from: https://academic.oup.com/jamia/article/22/e1/e141/703505/. Accessed July 2019. [cited Jul 2019]. 10.1093/jamia/ocu050. 10.1093/jamia/ocu050 PMC4428438 25769682

[8] The Scottish Health Research Register. Available from: www.registerforshare.org.uk. Accessed Oct 2019.

[9] McKinstry B, Sullivan FM, Vasishta S, Armstrong R, Hanley J, Haughney J, et al. Cohort profile: the Scottish Research register SHARE. A register of people interested in research participation linked to NHS data sets. BMJ Open. 2017;7(2). Available from: http://bmjopen.bmj.com/content/7/2/e013351.long. Accessed July 2019. [cited Jul 2019]. 10.1136/bmjopen-2016-013351. 10.1136/bmjopen-2016-013351 PMC5293989 28148535

[10] The All of Us Research Program Investigators. The “All of Us” Research Program. N Engl J Med. 2019;381(7):668–76. 10.1056/NEJMsr1809937. 10.1056/NEJMsr1809937 PMC8291101 31412182

[11] The Health Informatics Centre: University of Dundee. Available from: www.dundee.ac.uk/hic/patientrecruitment/. Accessed Oct 2019.

[12] Scottish Government. The use of the CHI (Community Health Index) to support integrated care across the NHS in Scotland. 2013. Available from: www.ehealth.scot/wp-content/uploads/documents/Health-and-social-care-CHI-Guidance-version-1-1-Strategy-Board-Approved-June-20131.pdf. Accessed July 2019. [cited Jul 2019].

[13] British National Formulary. Available from: https://bnf.nice.org.uk/. Accessed Oct 2019.

[14] Burton PR, Murtagh MJ, Boyd A, Williams JB, Dove ES, Wallace SE (2015). Data safe havens in health research and healthcare. Bioinformatics..

[15] Scottish Government. Charter for Safe Havens in Scotland: Handling Unconsented Data from National Health Service Patient Records to Support Research and Statistics. 2015. Available from: www.gov.scot/publications/charter-safe-havens-scotland-handling-unconsented-data-national-health-service-patient-records-support-research-statistics/. Accessed July 2019. [cited Jul 2019].

[16] Niedner CD (1990). The entity-attribute-value data model in radiology informatics. Proceedings of the 10th conference on computer applications in radiology.

[17] Chen RS, Nadkarni P, Marenco L, Levin F, Erdos J, Miller PL (2000). Exploring performance issues for a clinical database organized using an entity-attribute-value representation. J Am Med Inform Assoc.

[18] The Information Services Division of NHS National Services Scotland. Prescribing Information System [Internet]. Available from: www.isdscotland.org/Health-Topics/Prescribing-and-Medicines/Prescribing-Datamarts/docs/PIS_fields_for_researchers_v5_eDRIS%20Guidance.pdf. Accessed July 2019.

[19] Li B, Han L, Yin H, Tang K, Gao Y, Klawonn F, Lee M, Weise T (2013). Distance Weighted Cosine Similarity Measure for Text Classification. Intelligent Data Engineering and Automated Learning – IDEAL 2013. 8206.

[20] Hanley JA, McNeil BJ (1982). The meaning and use of the area under a receiver operating characteristic (ROC) curve. Radiology..

[21] Safari S, Baratloo A, Elfil M, Negida A (2016). Evidence based emergency medicine; part 5 receiver operating curve and area under the curve. Emerg (Tehran).

[22] Carpenter J, Bithell J (2000). Bootstrap confidence intervals: when, which, what? A practical guide for medical statisticians. Stat Med.

[23] Craswell N, Liu L, ÖZsu MT (2009). Precision at n. Encyclopedia of database systems.

[24] Zhang E, Zhang Y, Liu L, ÖZsu MT (2009). Average precision. Encyclopedia of database systems.

[25] Craswell N, Liu L, MT ÖZ (2009). Mean Reciprocal Rank. Encyclopedia of Database Systems.

