# Supplementary material for: Efficient identification of patients eligible for clinical studies using case-based reasoning on Scottish Health Research register (SHARE)
Source: BMC Med Inform Decis Mak. 2020 Apr 19;20:70. doi: 10.1186/s12911-020-1091-6 (PMC7169032; doi:10.1186/s12911-020-1091-6)
Supplement: Supplementary file 1 — Additional file 1. Summary of nine projects included for analysis. [file 12911_2020_1091_MOESM1_ESM.docx]

| **Summary of nine projects included for analysis** | | | | | |
| --- | --- | --- | --- | --- | --- |
| **Project Acronym**  **(Registration)** | **Project Working Title** | **Inclusion & Exclusion Criteria** | **No. Required** | **No. Identified** | **No. Recruited**  **(No. Identified in Database)** |
| ALPHA  (EU Clinical Trials Register: 2014-002305-38) | Does allopurinal reduce right ventricular mass in lung disease associated pulmonary hypertension? | Inclusion:   - Chronic obstructive pulmonary disease   Exclusion:   - Gout - On allopurinol - ICD 10 codes: C00-C97, D00-D09, D37-D48, K70.3, K70.4, K74, K74.0, K74.1,K74.2, K74.6, N18.3, N18.4, N18.5 - BNF codes: 8.2.1, 8.1, 3.1.3 | 72 | 1027 | 14(8) |
| ALLAY  (EU Clinical Trials Register: 2014-002083-33) | Does Allopurinol Regress Left Ventricular Hypertrophy in  Patients With Treated Hypertension? | Inclusion   - Age over 18 - Diagnosed with essential hypertension - On stable antihypertensive therapy for at least 3 months prior to trial screening - Screening ABPM (or home based BP monitoring) with daytime average systolic <135mmHg or 24-hour average systolic <= 130mmHg - Screening echocardiography based diagnosis of LVH based on ASE criteria( males >115g/m2, females >95g/m2)   Exclusion   - Intolerance to allopurinol - Left Ventricular Ejection Fraction <45% - On echocardiography screening (I50) - Severe aortic stenosis on echocardiography screening - Active gout (i.e. flare up <2yrs) or currently on allopurinol - Severe hepatic disease - Renal disease; CKD class 38 or worse (N18.3 N18.4 N18.5) - On azathioprine, 6 mercaptopurine, or theophylline - Malignancy (receiving active treatment) (C00 - C97) or other life threatening diseases - Pregnant or lactating women - Any contraindication to MRI (claustrophobia, metal implants, penetrative eye injury or exposure to metal fragments in eye requiring medical attention) - Have participated in other clinical trial of an investigational medicinal product within the previous   30 days   - Unable to give informed consent | 20 | 3361 | 9(9) |
| METFORMIN  (EU Clinical Trials Register: 2014-003189-26) | Metformin and its effects on Myocardial Dimension and Left Ventricular hypothophy in normotensive patients with Coronary Artery Disease | Inclusion   - Age over 18 - History of lschemic heart disease/coronary artery disease - Screening echocardiography based diagnosis of LVH based on American Society of Echocardiography criteria(male >=115g/m2,Female >=95 g/m2)   Exclusion   - Diabetes (ICD code E10- E14) AND/OR taking any antidiabetic medications (BNF 6.1.2) - Heart failure (I50) - Left ventricular ejection fraction <45% or with poor/moderate or severe systolic dysfunction (I50) - Contraindications to cardiac MRI (pacemakers, mechanical valves) (ICD code Z95.0) - Atrial Fibrillation (I48) - Pregnancy and lactating mother - Renal disease; CKD class 3B or worse(N18.3,N18.4, N18.5) - Have participated in any other clinical trial within the previous 30 days - Malignancy (receiving active treatment (C00 -C97) or other life threatening diseases | 35 | 54 | 5(0) |
| REFORM  (EU Clinical Trials Register: 2014-002742-42) | Research into the Effect of SGLT2 inhibition on left ventricular remodelling in patients with heart failure and diabetes Mellitus | Inclusion:   - Age: 18-75 - HF - Diabetes - On Diuretic   Exclusion:   - On Insulin therapy | 50 | 142 | 16(10) |
| IMPOCT  (Clinical trials.gov: NCT02586636) | Impact of OCT1 genotype and OCT1 inhibiting drugs on an Individual’s Tolerance of Metformin | Inclusion:   - Age:18-80 - White European - Able to complete the symptom severity score and Bristol stool chart independently - eGFR>60   Exclusion:   - Diabetes - Involvement in CTIMP within 30 days - Pregnancy or planning to conceive - Metformin history - Gastrointestinal pathology - Daily treatment with PPI, anti-spasmodic, or anti-motility drugs or OCT1 inhibiting drugs | 40 | 620 | 2(2) |
| TARDIS  (/) | Tracking Antimicrobial Resistance in respiratory DISease | Inclusion   - Age >40 - COPD   Exclusion   - Will be checked at visit | 150 | 487 | 21(21) |
| 4P  (ISRCTN registry: 12700399) | BNP for Personalised Primary Prevention in Diabetes | Inclusion   - Age 40-85 - Type 2 Diabetes mellitus (E11) - Blood pressure ≤140/80mmHg - HbA1c value ≤64mmol/mol   Exclusion   - Blood pressure ≥140/80mmHg - eGFR <60 (N18.3-18.6) - Atrial fibrillation (I48) - Heart failure (I42,43,50) - Peripheral vascular disease (I73-75) - Stroke (I63,65,66) - TIA (G45) - Ischaemic heart disease (I20-25) - Inability to consent (F70-79, G30) | 60 | 512 | 5(5) |
| HF  (/) | How do older Heart Failure patients manage their medication? | Inclusion   - Age over 70 years - ICD - I50 (Heart Failure) - NYHA class II to IV - Prescribed medication: BNF code 2.2.2 (frusemide) and 2.5.5.1 (ACE i)   Exclusion   - Living in nursing or residential home accommodation | 30 | 346 | 19(19) |
| Immunostat  (Clinical trials.gov: NCT02984293) | ImmunoStat | Inclusion:   - LILRB5 rs12975366 genotype of either T/T or C/C - Males aged 61-69 years or Females aged 40-69 - Not on statin   Exclusion:   - More than 3 prescriptions in last 12 months | 60 | 515 | 28(28) |
